# Expanding horizons and navigating challenges for enhanced clinical workflows: ChatGPT in urology

**DOI:** 10.3389/fsurg.2023.1257191

**Published:** 2023-09-07

**Authors:** Ali Talyshinskii, Nithesh Naik, B. M Zeeshan Hameed, Ulanbek Zhanbyrbekuly, Gafur Khairli, Bakhman Guliev, Patrick Juilebø-Jones, Lazaros Tzelves, Bhaskar Kumar Somani

**Affiliations:** ^1^ Department of Urology, Astana Medical University, Astana, Kazakhstan; ^2^ Department of Mechanical and Industrial Engineering, Manipal Institute of Technology, Manipal Academy of Higher Education, Manipal, India; ^3^ Department of Urology, Father Muller Medical College, Mangalore, India; ^4^ Department of Urology, Mariinsky Hospital, St Petersburg, Russia; ^5^ Department of Urology, Haukeland University Hospital, Bergen, Norway; ^6^ Department of Urology, National and Kapodistrian University of Athens, Sismanogleion Hospital, Athens, Marousi, Greece; ^7^ Department of Urology, University Hospital Southampton NHS Trust, Southampton, United Kingdom

**Keywords:** chatGPT, generative AI, healthcare, urology, workflow

## Abstract

**Purpose of review:**

ChatGPT has emerged as a potential tool for facilitating doctors' workflows. However, when it comes to applying these findings within a urological context, there have not been many studies. Thus, our objective was rooted in analyzing the pros and cons of ChatGPT use and how it can be exploited and used by urologists.

**Recent findings:**

ChatGPT can facilitate clinical documentation and note-taking, patient communication and support, medical education, and research. In urology, it was proven that ChatGPT has the potential as a virtual healthcare aide for benign prostatic hyperplasia, an educational and prevention tool on prostate cancer, educational support for urological residents, and as an assistant in writing urological papers and academic work. However, several concerns about its exploitation are presented, such as lack of web crawling, risk of accidental plagiarism, and concerns about patients-data privacy.

**Summary:**

The existing limitations mediate the need for further improvement of ChatGPT, such as ensuring the privacy of patient data and expanding the learning dataset to include medical databases, and developing guidance on its appropriate use. Urologists can also help by conducting studies to determine the effectiveness of ChatGPT in urology in clinical scenarios and nosologies other than those previously listed.

## Introduction

In this modern day and age medical practitioners are challenged with a significant amount of administrative tasks and documentation. Unfortunately, these duties frequently require more time to complete than actual medical procedures on patients (1). Sadly, the present healthcare system in most countries neglects to address the challenges faced by physicians and aide workers. Recent research exhibited that bureaucratic duties, inadequate pay for additional hours worked, and sporadic working hours were found to be detrimental associated factors identified by doctors (2). One worrying issue regarding doctors' well-being is job-related stress manifesting into a very concerning issue referred to as “burnout”. Amongst medical specialists, urologists appear to be most heavily afflicted by this problem. Research indicates that rates of reported burnout among urologists go up as high as 68% and 54% across America and Europe respectively, something that calls for prompt and effective measures from healthcare institutions globally (3). The need of the hour is thus to improve efficiency and optimize the workload on urologists. The potential applications for generative artificial intelligence (AI) within the context of healthcare are numerous. From facilitating doctors' workflows to enhancing patient interactions and providing decision-support tools, this exciting technology presents myriad possibilities (4). ChatGPT, developed by Open AI in San Francisco, CA, USA, is a widely accepted generative AI representative (5, 6). The literature's evident benefits and prospects of ChatGPT are complimented by controversial research, underscoring the lack of a thorough understanding of this technology's current state. Moreover, when it comes to applying these findings within urological contexts, well-thought-out studies have not been many (7). Thus, our primary objective is rooted in analyzing available works cited by scholars on this topic with a keen focus on delineating pertinent issues such as what aspects are beneficial or disadvantageous in using ChatGPT systems. Also if they are efficiently exploited by professionals specializing in fields such as urology.

## Overview of applications of ChatGPT in healthcare

**Figure 1:**
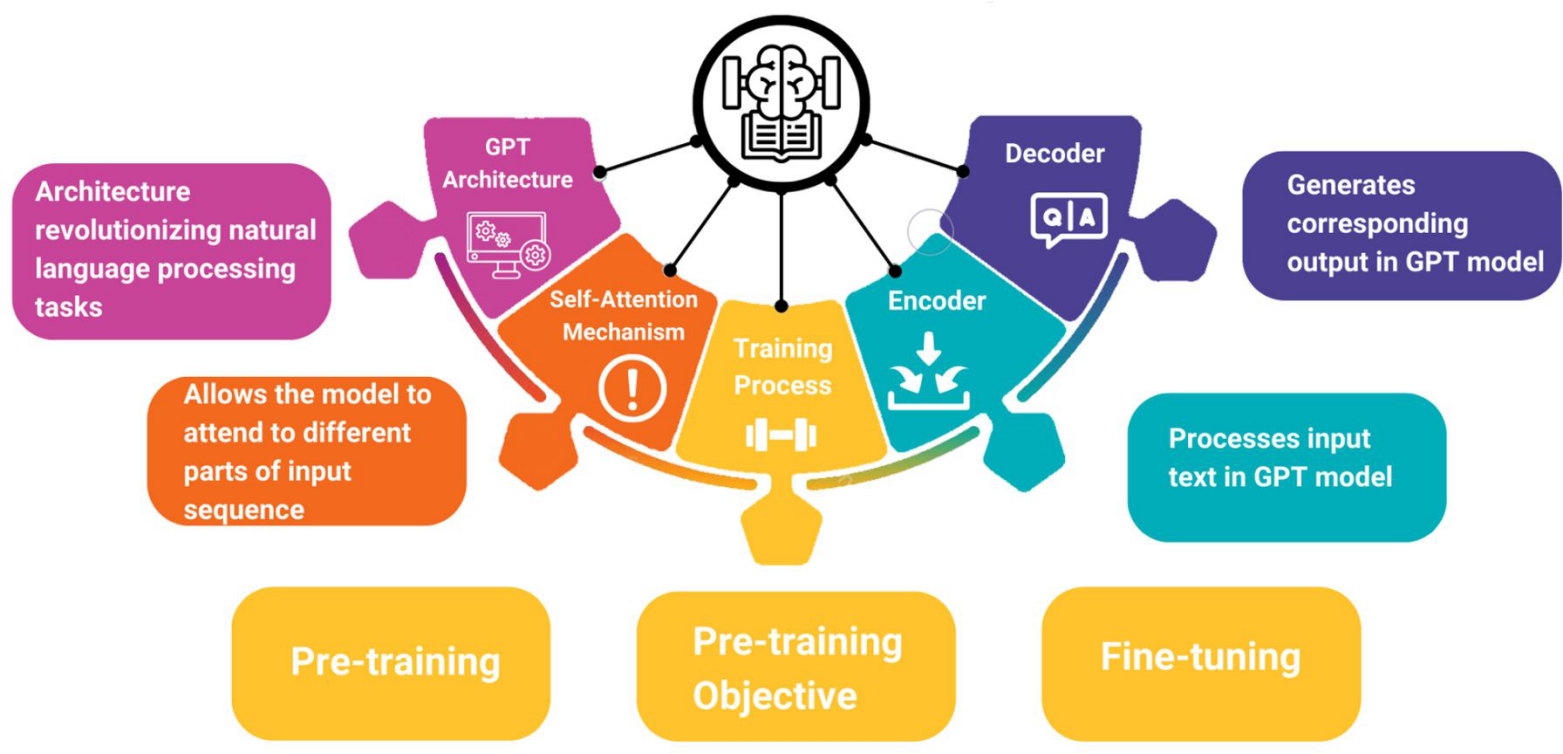
Overview of ChatGPT architecture and training process.

OpenAI established ChatGPT in November 2022 to construct conversational AI systems that can understand and respond to human language. Over its different iterations response accuracy and human likeness have been improved. ChatGPT's zero-shot learning allows it to respond coherently to novel inputs. Encoders and decoders comprise ChatGPT's transformer architecture. The transformer design relies on the attention mechanism, which lets the model focus on different parts of the input text while generating output (8). Figure 1 shows an overview of the ChatGPT architecture and the training process needed to process the input and deliver the output.

The potential implications of employing ChatGPT in various medical areas have been explored through numerous articles. Many useful insights are featured within the work of D'Amico et al. (9). They evaluated how ChatGPT can assist with neurosurgical health data collection and processing according to their logic and increasing efficiency among health professionals. Having such access will enable better quality patient monitoring by allowing them immediate access to historical patient records whenever needed. It can also help in creating a credible source for counseling self-help tips much like a therapist or physician and can get help in real-time during an emergency without any delay. ChatGPT assistance for decision-making was found to expedite sorting and prioritizing patients who have a pressing medical situation. ChatGPT can potentially provide patients with accurate information about various illnesses and related symptoms that may prevent unnecessary and premature appointments with the doctor. The remote sharing of medical information can contribute to lowering the burden of healthcare professionals by enabling remote contact between doctors and patients, thereby significantly reducing waiting times in the process.

Investigating advancements in emergency medical technology, Bradshaw (10) explored the implications of implementing ChatGPT in a medical context. By streamlining data input procedures through optimized automation, this innovative tool may save healthcare providers a significant amount of time. In addition to reducing instances where human error is possible, ChatGPT also offers clear benefits related to improved communication between physicians and patients, which ultimately results in greater levels of satisfaction overall.

In the field of clinical oncology, ChatGPT holds tremendous potential by maximizing patients' personalized information gathered from case histories and medical records (11). This technology streamlines screening processes while allowing healthcare practitioners to make informed judgments based on detailed patient-specific data analysis.

However, the opinion on ChatGPT immaturity in physicians' assistance also exists. Farhat (12) assessed ChatGPT's effectiveness in providing support for issues related to anxiety and depression, based on the chatbot's responses and cross-questioning. According to the findings, there were significant inconsistencies and ChatGPT's reliability was low in this specific domain. Cao et al. (13) reported that Six liver cancer specialists had found ChatGPT unreliable in answering 20 questions concerning monitoring and diagnosis. Inaccurate answers sometimes included inconsistent or deceptively comforting, if not erroneous, information about individual LI-RADS categories. Potential scenarios, where ChatGPT could be used with associated risks and benefits are briefed in Table 1.

**Table 1:** Current scenarios to use chatGPT in medicine, their potential advantages and shortcomings.

Domain	Feature	Potential advantages	Disadvantages
Clinical documentation and note-taking	Structuring of medical history	Potential for reduced time expenditure on these duties which implies more significant periods dedicated towards conversations with patients as well as treatment coursing; Enhanced collaboration between physicians and patients	Lack of contextual understanding; Misinterpretation of ambiguous inputs; limited clinical experience; Lack of personalized data privacy
	Medical history summarizing		
	Notes creation		
	Follow-up advices		
	Real-time documentation assistance		
	Decision support		
Patient communication and support	Self-evaluation of symptoms	Providing patients with reliable information pertaining to their health state, treatment alternatives available and foreseeable implications; Providing communication with physicians and high quality patient care for patients with language barriers; Alleviate patients’ emotional stress by acknowledging their concerns	Misunderstandings since patients may not clearly explain condition and write input; Inability to perform physical examination, estimate non-verbal signs, provide hands-on care; Lack of knowledge on recent advancements in healthcare; Lack of personalized data privacy; Lack of empathy
	Language barriers		
	Emotional support		
	Non-judgmentality		
	Confidentiality		
	24/7 availability and accessibility		
	Educational tool		
Medical education and preparation for medical entrance exams	Interactive education platform	ChatGPT has vastly knowledgeable user base caters to students and experts who can access various topics; Serves as reliable and dynamic platform for online learning experiences where it immediately analyses errors made by its users after each response attempt with advices for further improvement. As containing vast medical knowledge, can aid and help users when preparing for specific medical entrance exams	Knowledge are limited by 2021 year; The absence of a certified medical source training dataset; Variation in ChatGPT's medical test accuracy across different countries; Lack of clinical experience; Insufficient explanation;
	Knowledge in all medical disciplines		
	Real-time errors analysis		
	Advices on further education		
Literature review and research support	Generating completely original content	ChatGPT has proficient capacity for processing copious amounts of information quickly provides clients with succinct summaries; ChatGPT is able to simplify the process of preparing manuscript; It is possible to generate medical paper from scratch; It is possible to identify potentially fruitful research ideas through the clarification of problematic issues that require scientific analysis	Inaccurate references search; No up-to-date text generation; Inability in web search; The absence of a certified medical source training dataset; Lack of critical thinking; Risk of accidental plagiarism; Potential for loosing of analytical potentials by users;
	Correct manually-written references in various styles		
	Statistical data processing		
	Editing services for english-language texts		
	Brainstorming		

## Clinical documentation and note-taking

ChatGPT assumes a text-oriented strategy that can facilitate the management of medical data entry and note-taking processes based on individualized analysis of symptoms and test outcomes specific to patients. As a result of this, there is potential for reduced time taken on this aspect, which implies more time dedicated to patient conversation and counseling. More so, structuring intricate information consistently via the use of ChatGPT serves as a tool for reinforcing comprehension and the overall message among its users. In their research article, Singh et al. (14) point out the various abilities of ChatGPT, from generating ocular extracts to offering operational notes for healthcare providers. Based on these findings, ChatGPT has the potential to provide tailored prescription information, consultation time, and follow-up advice as appropriate. Additionally, Zhou et al. (15) indicated that the model can furnish an elaborate overview of medical history as well as the patient's current health status via test results analysis. What is more remarkable is that this model is knowledgeable enough to give sound clinical suggestions while presenting a summary report about a patient's current well-being, both grounded in its comprehensive database. Lastly, given its vast skillset and experience base thereof, doctors may avail themselves of real-time documentation assistance via ChatGPT.

## Patient communication and support

Patients using ChatGPT can get reliable information about their health, treatment alternatives available, as well as foreseeable implications, as demonstrated by Yeo et al. (16) indicating an impressive accuracy rate for ChatGPT knowledge on cirrhosis (79.1%) and HCC (74.0%). To complement the platform's capabilities, ChatGPT structures patient questions to aid in symptom evaluation and provides preliminary suggestions based on responses given by the patients themselves, ideas that can assist in establishing their symptom severity while also determining when they require emergency medical treatment or if self-care practices are sufficient (17).

Addressing language barriers is paramount in ensuring that high-quality patient care can be delivered, and one solution is the use of translation software. As reported by Yeo et al. (18) GPT-4 outperformed ChatGPTs response accuracy when answering questions in English, Korean, Mandarin, and Spanish. Moreover, sentimental support provided via ChatGPTs empathic dialogue can help alleviate patients' emotional stress by acknowledging their concerns while guiding them on managing their mental well-being. In an assessment of ChatGPT's ability to detect emotional subtleties using the Levels of Emotional Awareness Scale (LEAS), Elyoseph et al. (19) discovered that the chatbot performed significantly better than most humans during both initial and follow-up evaluations.

## Medical education

Optimizing medical education appears promising with the use of ChatGPT because its vastly knowledgeable user base caters to students and experts who can access various topics concerning this field. Oh et al. (20) attested to ChatGPT's efficiency in providing surgical teaching through its analysis of various responses submitted, resulting in a 76.4% accuracy percentage on tests administered by the Korean Board of General Surgery. Li et al. (21) reflected even better results when they scored this tool with an average score of 77.2% accuracy on virtual objective structured clinical exams administered within Singapore, surpassing human averages at a ratio of over 4% superiority. Also notable is that some human evaluators found it challenging to distinguish between replies from people and those from ChatGPT because of the program's smart learning capability. However, Alfershofert et al. (22) evaluated the performance of ChatGPT on six different national medical licensing exams and investigated the relationship between test question length and ChatGPT's accuracy. They discovered significant variation in ChatGPT's test accuracy across different countries, with the highest accuracy seen in the Italian exam (73 percent correct answers) and the lowest accuracy seen in the French exam (22% correct answers). Moreover, they discovered that queries requiring multiple correct responses, such as those on the French examination, presented a greater challenge to ChatGPT.

## Medical literature review and research support

ChatGPT continues to amaze the scientific community due to its exceptional capabilities in streamlining medical article composition and literature appraisal (23). Holly Els assessed ChatGPT's textual output and highlighted its exceptional performance regarding generating completely original content. A highly rated component was its ability to produce machine-generated texts that could even fool human reviewers in over a third of attempts during her test analysis (24). However, the opposite opinion also exists. As stated by Arif et al. (25) ChatGPT can be used as a supplement to constructive writing, examining information, and rephrasing the text rather than as a replacement for a complete original blueprint. Because medical literature is a constant process of updated research, there is growing worry that ChatGPT may now be easily utilized for authoring articles that may lack clinical reasoning and critical thinking.

In addition to generating the finished text using ChatGPT, it is also possible to simplify the process of preparing your manuscript. ChatGPT can quickly overwrite manually-written references in various styles, such as Vancouver, MLA, or Chicago, but not create those *de novo* (26). ChatGPT can function as a proficient biostatistician for statistical data processing, determining the most informative methods of statistical analysis, while also advising visual support (27). This advanced technology excels beyond the capabilities of commonly accessible translators, offering exceptional editing services for English-language texts at a C1 level of language proficiency (28).

This technology allows for not only the direct examination of the text but also the identification of potentially fruitful research ideas through clarification of problem-solving issues that require scientific analysis. Users can also chat with ChatGPT to discuss principal concepts and potential developments, promoting critical thinking among young professionals and motivating them to test certain hypotheses (29).

## Implications for urology practice

Several investigations have explored the application of ChatGPT in the domain of medical expertise and urological patient care. One study conducted by Tung et al. (30) involved using ChatGPT as a virtual healthcare aide for preoperative TURP concerns. The tool provided succinct yet reassuring responses regarding potential dangers along with encouraging individuals to seek input from expert physicians for additional clarification, while also offering post-operative relief by advising on identifying alarming symptoms and providing detailed guidance on physical activity as well as easing constipation.

In another inquiry carried out by Ilie et al. (31), researchers examined the role played by AI technology specifically through ChatGPT in medico-education settings. The reviewers interviewed ChatGPT to provide an overview of localized prostate cancer treatment plans and established that it was particularly reliable for delivering accurate medical information. However, its usage was primarily based on US data which could lead to such findings being slightly biased.

The prevention and screening of prostate cancer were explored by Zheng et al. (32) in evaluating the AI-powered system ChatGPT-4's effectiveness in offering advice on the matter through NCCN recommendations-based questions alongside clinical data points given to them. According to urologists involved with the research project, most of ChatGPT's responses were deemed appropriate. However, a few responses were not suitable or inaccurate underlining the need for exhaustive review before accepting AI-generated information unquestionably.

Another research paper conducted by Zhu et al. (33) analyzing several language models' capacities for addressing issues surrounding prostate cancer found that AI tools such as ChatGPT can be used effectively to provide patients with relevant information about screening procedures, prevention measures as well as treatment options, drawing insights from clinical expertise records alongside established patient educational standards. This facilitates informed decisions between doctors and their patients, ultimately empowering them with medical knowledge and allowing them to reach a shared decision making.

ChatGPT's proficiency in urology and its potential benefits for residents were observed by Deebel et al. (34). The American Urological Association (AUA) Self-Assessment Study Program ratings varied for ChatGPT. To broaden its educative scope, ChatGPT must increase its wealth of knowledge. Additionally, Schuppe et al. (35) utilized AI-based writing support from ChatGPT to draft a Nelson syndrome case study post-bilateral adrenalectomy. In this way, ChatGPT assisted in outlining, developing, and concluding the case study. As mentioned earlier, in every aspect of the application of ChatGPT, there is both confirmation and refutation of the usefulness of the technology. Medical Education is not an exclusion. Huynh et al. (36) evaluated the utilization of ChatGPT as an educational supplement for urology trainees and practicing physicians in the American Urological Association Self-assessment Study Program. ChatGPT correctly answered 36/135 (26.7%) open-ended questions and 38/135 (28.2%) multiple-choice questions. Indeterminate replies were obtained in 40 (29.6%) of the cases and in 4 (3.0%). Although regeneration reduced uncertain replies, it did not raise the number of accurate responses. ChatGPT gave consistent reasons for erroneous responses and remained concordant between correct and incorrect answers for open-ended and multiple-choice questions. The same opposite results were found by Whiles et al. (37) When evaluating ChatGPT's ability to provide patient counseling answers based on clinical care recommendations in urology. The authors stated that when evaluating healthcare-related recommendations from present AI models, users should exercise caution. Additional training and changes are required before these AI models can be trusted by patients and doctors. Also, Misheyev et al. (38) characterized the information quality and detected misinformation regarding prostate, bladder, kidney, and testicular malignancies from four AI chatbots: ChatGPT, Perplexity, Chat Sonic, and Microsoft Bing AI. The results indicate that AI chatbots produce information that is generally accurate and of moderate to high quality in response to the top urological malignancy-related search queries. However, the responses lack clear, actionable instructions and exceed the recommended reading level for consumer health information.

## Challenges and risks of using ChatGPT in healthcare

An analysis of ChatGPT limitations should come first before explicating further the positive aspects, especially because our understanding of them might be incomplete.

One primary challenge facing ChatGPT is its lack of web crawling capabilities which currently limits access solely to information acquired before 2021. Ayoub et al. (39) conducted a cross-sectional analysis to evaluate ChatGPT's capabilities as a source of medical knowledge, using Google Search as a comparison, and discovered that ChatGPT performed better than Google Search when providing general medical knowledge, but worse when providing medical recommendations. Manolitis et al. (40) assessed the efficacy of a ChatGPT API 3.5 Turbo model to a standard model in supporting urologists in getting precise, reliable medical information. The API was accessed using a Python script written particularly for this study and based on 2023 EAU guidelines in PDF format. This custom-trained model provides clinicians with more exact, rapid responses concerning specific urologic issues, thereby assisting them in providing better patient care rather than the existing standard model.

Using deceptive or inaccurate data to train, ChatGPT could also pose a significant risk, leading to inconsistent or untrue medical responses. Tung et al. (34) observed that ChatGPT gave inaccurate information, such as a percentage risk of retrograde ejaculation based on current research. ChatGPT did not offer clarifying questions to improve diagnosis, and replies were also inconsistent. Skewed training data can result in skewed output, and excessive reliance on ChatGPT can reduce patient adherence and promote self-diagnosis. To ensure the accuracy, validity, and reliability of ChatGPT-generated content, rigorous validation and ongoing updates based on clinical practice are necessary.

“Hallucination” in writing, where it is influenced more by learned patterns rather than scientific facts, is what leads to these mistakes (41). Generative ChatGPT can show signs of this phenomenon due to being trained on large amounts of unsupervised data. Farhat et al. (42) assessed the performance of ChatGPT in creating an abstract and references for bibliometric analysis. Despite the well-written quantitative data display, ChatGPT offered incorrect information regarding major authors, countries, and avenues. Moreover, ChatGPT provided either non-existent or unrelated to the study references. When ChatGPT was questioned about the sources, it apologized and provided a fresh set of references, however, the references were similarly non-existent following further inquiry. These data show that ChatGPT is configured to react to any enquiry, regardless of correctness, and it accepts no responsibility for any inaccuracies. Summarizing the above, the following ChatGPT-associated intrinsic issues can be distinguished: hallucination, biased content, not real-time, misinformation, and inexplicability. Some authors proposed adaptive steps to combat them. So, Sohail et al. (8) discussed that algorithmic improvement, inputting the queries properly, verifying generated responses, and human feedback, and refining the training data to remove or mark the biased content might help overcome these problems.

ChatGPT can be a valuable tool for literature review and research. Nevertheless, we must recognize its limitations since it cannot replace human critical thinking, knowledge acquisition, or peer review processes (43). Presumably using AI technology may have serious unintended consequences, leading young scientists especially into losing their analytical potential over time. Additionally “knowledge homogenization” could result if every individual merely receives data from an unbiased “collective consciousness” without any supervision exercised (44). Generative AI researchers like ChatGPT risk accidental plagiarism while carrying biases, emphasizing the need for responsible ethical conduct on their part. Finally, it is important to mention that ChatGPT fails to meet either GDPR or HIPAA standards, creating issues regarding safeguarding patient health information (PHI) and personal data. As stated by Cacciamani et al. (45) patient safety, cybersecurity, transparency and interpretability of the data, inclusivity and equity, fostering responsibility and accountability, and the preservation of providers' decision-making and autonomy are among the potential ethical issues that must be taken into account when implementing AI in clinical practice.

While the majority of the medical community's concentration is on ChatGPT, other Large Language Models (LLMs) should be kept in mind and investigated to determine whether ChatGPT's shortcomings are unique or shared by the entire LLMs industry. Dao (46) compared ChatGPT, Microsoft Bing Chat, and Google Bard using the VNHSGE (Vietnamese High School Graduation Examination) dataset. The performance of BingChat, Bard, and ChatGPT (GPT-3.5) is 92.4%, 86.4%, and 79.2%, respectively, confirming the increased accuracy with BingChat use in English language education due to the incorporation of up-to-date information.

However, when it comes to the medical field, obvious advantages become hidden. Agarwal et al. (47) compared the applicability of ChatGPT, Bard, and Bing in generating reasoning-based multiple-choice questions (MCQs) for undergraduate students on the subject of physiology and found that BingChat generated significantly the least valid MCQs, while ChatGPT generated significantly the least difficult MCQs. Rahsepar et al. (48) compared the accuracy and consistency of responses generated by ChatGPT, Google Bard, and non-expert questions related to lung cancer prevention, screening, and terminology and found that Although ChatGPT had higher accuracy in comparison with the other tools, neither ChatGPT nor Google Bard, Bing, or Google search engines answered all questions correctly and with 100% consistency.

Thus, it is evident that the issues associated with the use of ChatGPT reflect the state of LLMs in general, emphasizing the need to improve all publicly accessible ChatBots powered by generative AI.

## Future directions and opportunities for research

As ChatGPT hinges on the data it obtains, certain key details must be manually inputted. However, potential advancements may allow for ChatGPT to independently extract data from digital archives sans human guidance (49). Additionally, the training database should be up-to-date and include relevant guidelines, as opposed to being limited to the year 2021 as it is currently. This strategy will equip ChatGPT with the necessary skills and reduce the likelihood of patients and medical students receiving incorrect information. Indeed, training with clinical guidelines significantly improves the accuracy of ChatGPT responses, as was confirmed by Manolitis et al. previously (40). UroChat (https://urochat.streamlit.app) was recently developed using the GPT 3.5-turbo model and 2023 EAU Guidelines. The presence of such chatbots is already a solution to several of ChatGPT's limitations. Nevertheless, future studies are needed to estimate its value for clinical decision-making, medical education, and patient counseling.

The potential of ChatGPT in aiding personalized therapy is considerable. Temsah et al.'s study indicates that by integrating the findings from the extensive global burden of disease research with advanced AI via open AI chat and utilizing the power of conversational ChatGPT-4, healthcare planning could be transformed at an individual level. With such integration, medical practitioners will have an improved ability to develop specially designed treatment plans based on patient's specific lifestyles and preferences (50).

The progress in AI has brought transformative benefits across various human endeavors, and scientific research is no exception. However, we must acknowledge potential risks from certain AI innovations like ChatGPT, specifically regarding fraudulent use, that may pose threats to scientific integrity. We must therefore take necessary measures and precautions against any emerging types of deceit linked with ChatGPT. Amongst current approaches include building diversified analytical tools capable of detecting instances of potentially fraudulent text produced through platforms like ChatGPT. Despite this approach, it is important to note an ongoing debate on ethical issues surrounding the extensive use of ChatGPT for purposes such as enhancing writing efficiency vs. interfering with original scientific inquiry.

Although banning ChatGPT might seem like a quick and easy solution, such actions could thwart progress in today's rapidly-evolving world. Instead, researchers must prioritize ethical considerations and aim for academic rigor despite any obstacles they face. Plagiarism can be avoided by refraining from copying and pasting unattributed content generated with AI tools into manuscripts. Prohibiting these instruments completely isn't necessary, it would be sufficient to simply document their usage within acknowledgments or methods sections when publishing research work, as stated by a recent article from Nature (51). Furthermore, credit should not be given to AI tools since they do not contribute to research outcomes but instead support revisions for original works only (45).

## Conclusion

Despite the many advantages offered by ChatGPT, it is not puzzling as to why urologists have yet to adopt this technology in their clinical and academic practice. The existing limitations mediate the need for further improvement of ChatGPT. These include measures such as algorithmic improvement, verifying generated responses, human feedback, refining the training data to remove or mark the biased content, ensuring the privacy of patient data, and developing guidance on its appropriate use to provide honest and reliable use of ChatGPT. Moreover, to determine the effectiveness of ChatGPT in urology, further studies in clinical scenarios and nosologies other than those previously listed are needed.

### Key points

• ChatGPT has emerged as a potential tool for facilitating doctors’ workflows.
• Despite the benefits of ChatGPT, several of its drawbacks, such as the lack of web crawling, the risk of accidental plagiarism, and concerns about patient data privacy, limit its reliable use.
• Studies on ChatGPT's potential in urology have not been many and are mainly focused on virtual healthcare aides for benign prostatic hyperplasia concerns, educational and prevention tools for prostate cancer, educational support for urological residents, and as an assistant in writing urological papers.
• Further improvements to ChatGPT should encompass the privacy of patient data, the possibility of independently extracting data from digital archives without human guidance, including medical databases, and the development of guidance on its appropriate use.
